# Predictors of self-management in patients with chronic low back pain: a longitudinal study

**DOI:** 10.1186/s12891-022-05933-2

**Published:** 2022-12-07

**Authors:** A. Banerjee, P. Hendrick, H. Blake

**Affiliations:** 1 Keele University, School of Allied Health Professions, Staffordshire, ST5 5BG UK; 2 Nottingham CityCare Partnership CIC, Nottingham, UK; 3 University of Nottingham, School of Health Sciences, Nottingham, UK; 4 NIHR Nottingham Biomedical Research Centre, Nottingham, UK

**Keywords:** Low back pain, Chronic low back pain, Self-management, Longitudinal study, Regression analysis, Predictors, Health education impact questionnaire

## Abstract

**Background:**

Self-management (SM) is a key recommended strategy for managing chronic low back pain (CLBP). However, SM programmes generate small to moderate benefits for reducing pain and disability in patients with CLBP. The benefits of the SM programme can potentially be optimised by identifying specific subgroups of patients who are the best responders. To date, no longitudinal study has examined the predictive relationships between SM and biopsychosocial factors in patients with CLBP. The aim was to determine whether biopsychosocial factors predict SM and its change over time in patients with CLBP.

**Methods:**

In this multi-centre longitudinal cohort study, we recruited 270 working-age patients with CLBP (mean age 43.74, 61% female) who consulted outpatient physiotherapy for their CLBP. Participants completed self-reported validated measures of pain intensity, disability, physical activity, kinesiophobia, catastrophising, depression and SM at baseline and six months. SM constructs were measured using eight subscales of the Health Education Impact Questionnaire (heiQ), including Health Directed Activity (HDA), Positive and Active Engagement in Life (PAEL), Emotional Distress (ED), Self-Monitoring and Insight (SMI), Constructive Attitudes and Approaches (CAA), Skill and Technique Acquisition (STA), Social Integration and Support (SIS) and Health Service Navigation (HSN). Data were analysed using General Linear Model (GLM) regression.

**Results:**

Physical activity and healthcare use (positively) and disability, depression, kinesiophobia, catastrophising (negatively) predicted (*p* < 0.05, R^2^ 0.07–0.55) SM constructs at baseline in patients with CLBP. Baseline depression (constructs: PAEL, ED, SMI, CAA and STA), kinesiophobia (constructs: CAA and HSN), catastrophising (construct: ED), and physical disability (constructs: PAEL, CAA and SIS) negatively predicted a range of SM constructs. Changes over six months in SM constructs were predicted by changes in depression, kinesiophobia, catastrophising, and physical activity (*p* < 0.05, R^2^ 0.13–0.32).

**Conclusions:**

Self-reported disability, physical activity, depression, catastrophising and kinesiophobia predicted multiple constructs of SM measured using the heiQ subscales in working-age patients with CLBP. Knowledge of biopsychosocial predictors of SM may help triage patients with CLBP into targeted pain management programmes.

**Trial registration:**

The study protocol was registered at ClinicalTrials.gov on 22 December 2015 (ID: NCT02636777).

**Supplementary Information:**

The online version contains supplementary material available at 10.1186/s12891-022-05933-2.

## Background

Low back pain (LBP) is a common condition (point-prevalence 18.3% ± 11.7%) [1], experienced by individuals of all ages globally [2]. It is the leading cause of disability, measured using the Years Lived with Disability (YLDs) [3, 4]. The high prevalence (18.3%), poor remission (54–90%) and high recurrence rates (24–80%) of low back pain [1, 5] result in chronic low back pain (CLBP) requiring higher health care needs such as general practitioner consultations found to be double that of matched controls without CLBP, and higher direct treatment cost estimated at £1000 per year per patient with CLBP [6]. In the United Kingdom (UK), national guidelines [7–9] recommend supported self-management (SM) as a management strategy in patients with CLBP.

The term SM is often inconsistently defined [10] as there is no agreed definition [11]. Nakagawa-Kogan [12] and colleagues defined SM as a combination of biological, psychological and social intervention techniques to alter long-term conditions by retraining self-regulating body processes to maximise disease management. This SM definition was based on the process model of therapy [13], which included role restructuring, forming the therapeutic alliance, developing commitment for change, analysing behaviour, negotiating treatment objectives, executing treatment, maintaining motivation, monitoring progress, and generalisation and termination of treatment. Clark defined SM as day-to-day home-management tasks to minimise the impact of disease as guided by healthcare providers [14], which highlighted both social and cognitive SM [15]. The UK National Health Service views SM as the ‘actions taken’ by individuals to recognise, treat and manage health and disease independently and in partnership with the healthcare system [16]. SM is advocated in the UK to manage long-term conditions, including low back pain [17].

For the purpose of this study, SM defines a dynamic and continuous ability to manage the disease, its symptoms, treatment, physical, psychological, and lifestyle changes [11] when living with a chronic illness. SM encompasses behaviour, role and emotional management with managing the disease by solving day-to-day problems, making conscious decisions, using appropriate health care resources, forming patient and healthcare provider partnerships and taking appropriate actions towards a healthy lifestyle [18]. SM programmes commonly consist of interdisciplinary group education delivered through a wide range of learning strategies in face-to-face, group-based, or internet-based interventions delivered by professionals or expert patients [18, 19]. The primary aim of SM programmes is to enhance SM and self-efficacy (confidence in one’s ability for SM) by utilising adult learning principles, case management theory and individualised treatment [20], allowing and encouraging individuals to manage their long-term conditions [21].

SM programmes are successful in reducing pain intensity [standardised mean difference- (SMD) -0.29 immediate in nine studies, -0.20 in long-term in four studies] and disability (SMD -0.28 immediate in nine studies, -0.19 in long-term in four studies) in patients with CLBP [22]. A similar reduction of pain intensity (11 studies, SMD -0.10) and disability (eight studies, SMD -0.15) has been reported when SM programmes have been delivered by expert patients or lay leaders [21]. However, at best, the clinical benefits of SM programmes are small to medium and often short-term in managing pain, disability, and self-efficacy in patients with CLBP.

These minor benefits of SM programmes are potentially due to several factors, including the lack of targeted SM interventions for specific populations [23]. The effectiveness of a treatment strategy depends on causal and mediation effects [24] and treatment matching [25]. Therefore, further understanding of the predictive relationships between SM constructs and biopsychosocial constructs in patients with CLBP may help identify a specific sub-group of patients with CLBP that may benefit from SM programmes and enhance the overall programme effectiveness. However, to our knowledge, predictors of SM in people with CLBP have only been investigated in one study to date, [26], which demonstrated that age [β =  − 0.197, Standard Error (SE) = 0.074] and poor overall health (negatively) and education attained at college. SM support (positively) predicted SM in 230 patients with CLBP when measured using the Patient Activation Measure (PAM) (β = 2.292, SE = 0.965). Yet, these predictive associations did not include psychological characteristics as potential predictors in the previous study and little attention has been paid to biopsychosocial measures and SM of CLBP. The aim of this study was to investigate whether there is a predictive relationship between SM constructs and biopsychosocial measures in patients who were seeking outpatient physiotherapy treatment for their CLBP.

## Methods

### Study design and sample size

This multi-centre prospective (non-experimental) longitudinal cohort study obtained a favourable ethical opinion from National Health Service Research Ethics Committee (Ref No 15/ES/1067- November 2015) and was conducted in line with the registered protocol (ClinicalTrials.gov ID: NCT02636777) [27]. A priori sample size calculation (using G*Power version 3.1.5 software) estimated that at least 130 participants would be required to detect a change with an effect size of 0.5 with 80% power and 5% significance level using the Health Directed Activity (HDA) subscale because this sub-scale produced the largest sample size required [28].

### Inclusion and exclusion criteria

Patients with CLBP were recruited from six UK National Health Service Trusts, including five acute care trusts and one community musculoskeletal service provider. For the study, low back pain was defined as pain in the posterior aspect of the body between the lower margins of the twelfth ribs and the gluteal folds with or without pain in one or both legs [5]. Patients, who were walking in the community without any aids, aged between 18 and 65 years, who attended outpatient physiotherapy for their chronic low back pain, and who could read, write, and understand English, were included in the study. Patients were excluded if they were diagnosed with cancer or other known or self-reported specific causes for their low back pain (major trauma, fracture, inflammatory condition, ankylosing spondylitis, Grade 3 and 4 spondylolisthesis, severe spinal canal stenosis, or lumbar intervertebral disc protrusion or extrusion, spinal deformity); had undergone spinal surgery in the last one year or scheduled for any major surgery in the coming six months; who were pregnant women or women who had childbirth in the previous one year; had cognitive impairment and neurological diseases; and had severely impaired vision and hearing hindering survey completion.

## Measures

### Biopsychosocial factors

LBP duration, presence of related leg pain, age, gender, ethnicity, postcode, educational level, current employment status, annual household income, marital status, and living arrangements were recorded at the baseline. Additionally, the amount and nature of treatment received, and analgesics used were collected at baseline and follow-up. Other biopsychosocial measures utilised in this study included the 11-item Numeric Pain Rating Scale [29], 24-item Roland Morris Disability Questionnaire [30], International Physical Activity Questionnaire-Short Form (IPAQ-SF) [31], Tampa Scale of Kinesiophobia (TSK) [32], Pain Catastrophising Scale (PCS) [33], Patient Health Questionnaire-9 (PHQ-9) [34].

### Self-management

Self-management was measured using a multi-domain scale Health Education Impact Questionnaire (heiQ) version 3 [35]. The heiQ consists of 40 items, which measure eight different constructs of SM: Health-Directed Activities (HDA), Positive and Active Engagement in Life (PAEL), Emotional Distress (ED), Self-Monitoring and Insight (SMI), Constructive Attitudes and Approaches (CAA), Skill and Technique Acquisition (STA), Social Integration and Support (SIS) and Health Service Navigation (HSN). Each of the 40 items can be scored using four-point ordinal scale options from ‘strongly disagree’ to ‘strongly agree’ with no neutral option given. The heiQ has high internal consistency (Cronbach’s a 0.70–0.89) and discriminant validity in patients with chronic diseases [28, 35]. The heiQ scale has been chosen for its ability to capture multiple SM constructs across physical, psychological and social domains and low response bias [36].

### Procedures

Willing patients completed an expression of interest and were screened using the study selection criteria. Eligible and consenting patients were requested to complete the surveys at two-time points: baseline and follow-up after six months. Responses could be completed using paper, online, and telephone survey modes at participant preference to maximise patient convenience and the survey response rate [37]. The Bristol Online Survey (BOS) platform was used for the online survey, which ensured data integrity and adhered to high data security standards.

### Data analyses

Data analyses were performed with a significance set at *p* < 0.05 in statistical software [IBM SPSS Statistics for Windows, Version 24.0 (Armonk, NY: IBM)]. Data were screened using stem-and-leaf plots and summaries to identify the presence of an impossible value. Scatter plots were visually assessed for any outliers. As the sample size was large (*n* > 100), normality was assessed using histograms and Q-Q plots. In the case of non-symmetrical or non-normal distribution, a Shapiro–Wilk test was utilised [38] for normality and Levene’s test for homogeneity of variance. The bootstrapped and accelerated intervals (*n* = 1000) were reported for all analyses.

The correlation between the model variables and each SM construct was estimated. Model variables that showed significant (*p* ≤ 0.05) correlation with the SM constructs were utilised in regression analysis. A multivariate regression analysis using a general linear model (GLM) was performed for each SM construct to identify predictors for baseline and change variables. Multi-collinearity was assessed using the Variance Inflation Factor (VIF < 10) for each independent variable.

## Results

### Characteristics of the patients

**Fig. 1:**
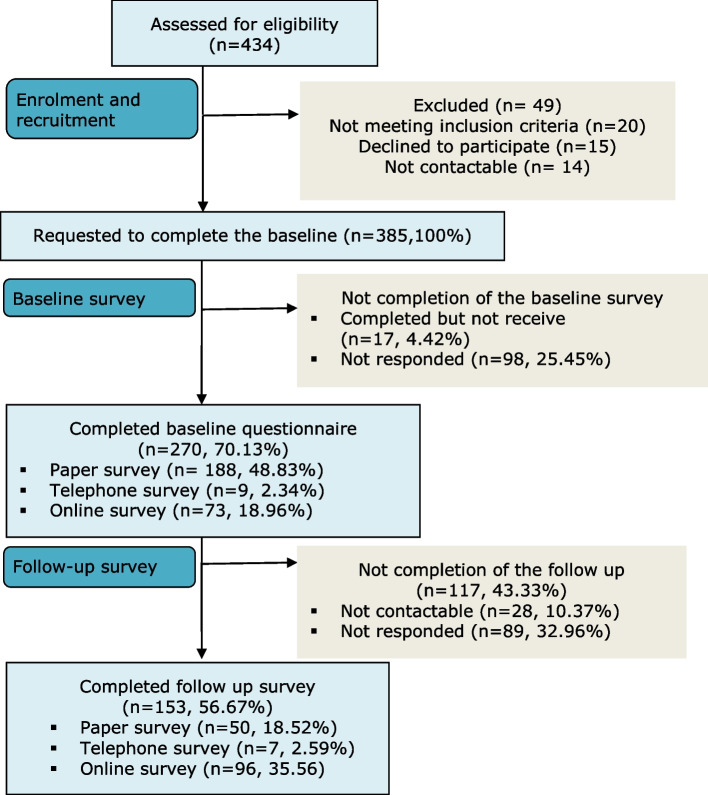
Flow of the participants in the study

A total of 434 patients with chronic low back pain (CLBP) expressed an interest in the study from six recruitment sites. Forty-nine (*n* = 49, 11.29%) patients were excluded at the screening stage for the following reasons: not meeting the inclusion criteria (*n* = 20, 4.61%), declined to participate (*n* = 15, 3.46%) and not contactable (*n* = 14, 3.23%). The remaining willing patients (*n* = 385) were invited to complete the baseline survey. Of these, 270 completed the baseline survey (Fig.1), and 153 completed the six-month follow-up survey. Recruitment of 270 participants was sufficient to detect a change of 0.4 (effect size) at baseline; completion of 153 six-month surveys was sufficient to detect a change of 0.5 (effect size) at follow-up.

The mean age of recruited patients was 43.74 years (standard deviation 11.89, *n* = 270). 61% of patients were female, and 83.7% of patients were from White ethnic backgrounds. The demographic details and clinical characteristics are presented in Supplementary File 1, showing no significant difference between completers and non-completers of the follow-up survey, except for the highest level of education. There was no significant difference (*p* < 0.05) in SM constructs at baseline between the recruitment centres. The bivariate correlation (Spearman's rho) between the eight SM constructs ranged from 0.15 to 0.59, suggesting they are related but separate SM sub-constructs. Demographic characteristics of the participants at baseline and comparison between completers and non-completers of the follow-up survey are presented in the Supplementary File.

### Predictors of self-management

**Fig. 2:**
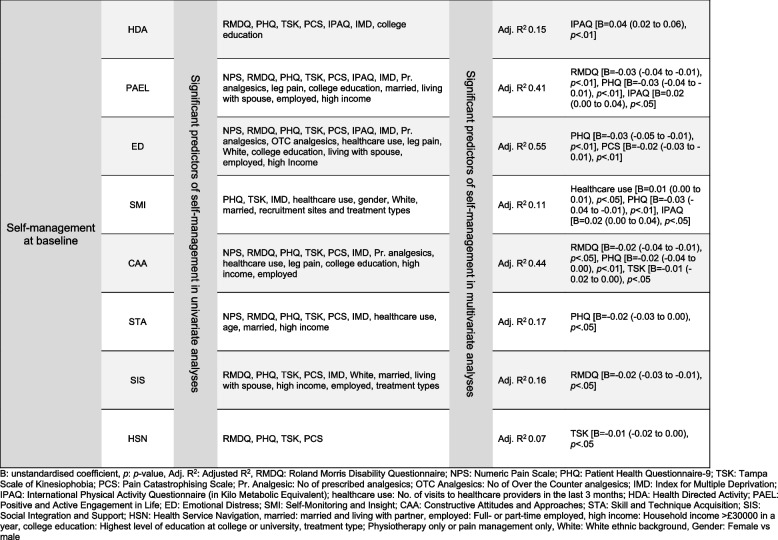
Predictors of self-management constructs at baseline

**Fig. 3:**
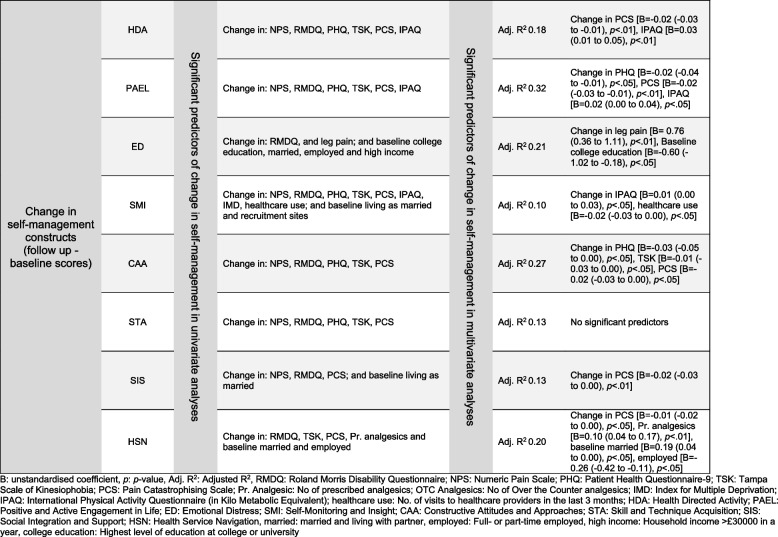
Predictors of change in self-management constructs at the follow up

Figure 2 summarises the regression results for the eight SM constructs at baseline. These analyses met the normality and homogeneity assumptions except for minor heteroscedasticity for HDA. For example, IPAQ was a significant predictor of HDA, and HDA increased by 0.04 for each Kilo metabolic equivalent (MET) increase in physical activity [F (7,260) = 7.70, *p* < 0.01] with an adjusted R^2^ of 0.15. Figure 3 summarises regression results for change in the eight SM constructs. For example, change in kinesiophobia and physical activity predicted change in HDA [F (6,139) 6.18, *p* < 0.01] with an adjusted R^2^ of 0.18.

Descriptive statistics of and correlations between the SM constructs, sensitivity analyses using mean substitution and baseline observed carried forward data imputations for lost to follow-up cases are presented in the Supplementary File.

## Discussion

We found that physical disability, physical activity levels, depression, kinesiophobia and catastrophising are the main modifiable biopsychosocial predictors of SM and its change in patients with CLBP. Further, we also found that age, pain intensity and pain duration do not predict SM and its change. Pain intensity and duration not predicting SM agrees with previous research [26]. However, our finding that age is not a predictor of SM contrasts with a cross-sectional study [26], where age correlates with SM negatively.

In our study, perceived physical disability negatively predicted three out of the eight SM constructs. However, physical disability measured using the Oswestry Disability Index was not a significant predictor of SM measured using the PAM in 230 patients with CLBP [26]. This difference in the findings could be due to the populations and different scales to measure SM and disability. For example, Kawi measured SM using PAM, which measures only patients’ activation and engagement from primary care and specialist pain centre in the USA.

We found that baseline depression had a significant negative predictive association for five out of eight SM constructs, suggesting that lower mood (i.e., symptoms of depression) was associated with poorer self-management outcomes. Although depression has not been examined as an explanatory variable in previous research investigating predictors of SM in patients with CLBP [26], depression is common in patients with other long-term conditions and has been found to impact negatively on SM. For example, depression is common in diabetes mellitus [39] and is an established negative predictor of diabetes SM in children [40] and adults [41, 42]. Depression has also been identified as a predictor of SM in older adults (*n* = 3292) in the UK, albeit using a different outcome measure [43]. Depression significantly predicted SM, measured using the Skill and Techniques Acquisition (STA) subscale of the German version of the heiQ, in patients with chronic conditions (*n* = 580), including rheumatism, asthma, orthopaedic disorders and inflammatory bowel disease [44].

Therefore, our results suggest that depression is a key predictor of certain constructs of SM in patients with CLBP, which agrees with broader research in patients with diverse long-term conditions.

Kinesiophobia and catastrophising have not previously been investigated as predictors of SM in patients with CLBP. However, distress and/or anxiety were investigated as a predictor of SM in patients with diabetes [45, 46]. An earlier study by Albright et al. [46] found stress had a significant negative predictive association with exercise and diet SM in 392 type II diabetes patients. Similarly, Schinkus et al. [45] found distress (measured using Diabetes Distress Scale) and anxiety (measured using the State-Trait Anxiety Inventory) were significant predictors of overall diabetes SM (measured using the Diabetes Self-Management Questionnaire) in 146 patients with type-I and type-II and gestational diabetes. These studies highlight the importance of measuring distress or anxiety or related variables as an explanatory variable in SM predictor studies.

In the present study, healthcare use, measured using the self-reported number of sessions attended at the general practitioner, physiotherapist, specialist, and other practitioners for CLBP, significantly predicted the SMI construct of SM. Further, education, income, living arrangements, being employed, being married, high annual income (> £30,000) and white ethnicity had significant associations in univariate GLM analysis. These results agree with the previous cross-sectional study [26], where age, education and income were significant predictors of SM in patients with CLBP. However, no significant predictive association was found in the multivariate GLM analysis for demographic and socioeconomic factors, suggesting that change in SM does not depend on age, education, and income.

Changes in depression, kinesiophobia, catastrophising, and physical activity level similarly predicted SM changes (R2 10% and 32%). Change in catastrophising predicted change in five out of eight SM constructs (HDA, PAEL, CAA, SIS and HSN). Catastrophising is a negative predictor for patients with CLBP and might contribute to the delayed recovery [47]. Patients with CLBP who had higher catastrophising were associated with a significantly higher disability using Roland Morris Disability Questionnaire in a UK population at a 12-month follow-up [48]. Further, patients with CLBP reported fluctuating negative pain-related thoughts affecting their coping and pain-related meta-cognition in a recent qualitative study [49], which could potentially influence HDA, PAEL, CAA, SIS and HSN. Change in depression predicted change in PAEL and CAA. Similarly, change in depression predicted SM in patients with diabetes [42] and long-term conditions [44].

### Theoretical underpinning

According to the Social Cognitive Theory, one of the critical theories underpinning SM, cognitive factors and psychological states modify self-judgement and the SM [50, 51]. Therefore, depression, excessive negative pain-related emotions or catastrophising and fear related to pain or re-injury or kinesiophobia may influence one's SM ability. Similarly, physiological states, including depression, kinesiophobia and catastrophising, influence self-efficacy and SM [51]. Therefore, along with promoting healthy living and physical activity [52], psychological and behavioural factors should be targeted to enhance SM in patients with CLBP. From a behaviourist point of view, capability, opportunity, and motivation interact to generate behaviour, in which capability includes one's physical and psychological abilities to engage in (SM) activity. So, SM programmes can utilise the Behaviour Change Wheel to create opportunities using interventions and policies to motivate individuals to change their capability [53].

### Strength and limitations

To our knowledge, this is the first prospective multi-centre longitudinal cohort study investigating predictors of SM in patients with CLBP. A strength of the study is the use of a multi-construct SM scale which provides a comprehensive assessment of SM constructs, and multi-component measures have not been used in previous studies of CLBP or other chronic conditions. The study has some limitations. The attrition rate was relatively high, with 117 patients lost to follow-up (43.33% attrition, compared with an anticipated 30%). However, there was no difference in baseline disease-related and SM outcomes between patients that completed follow-up and those who did not. The study had a poor representation of the Asian and male gender. However, it has been found that women are more likely to participate in survey research [54]. The lack of ethnic diversity may be due to excluding patients without good English language abilities due to a lack of funding for interpreters. However, the figures for ethnicity are comparable to the UK 2011 Census data [55] proportion of White and working citizens. Future studies may focus on ways to improve SM in low-income, non-White populations with CLBP, particularly individuals with poor health literacy.

## Conclusions

Conducting the first prospective longitudinal study investigating biopsychosocial predictors of SM, we conclude that disability, physical activity, depression, catastrophising, and kinesiophobia predict SM and its change in working-age adults who attended physiotherapy for their CLBP. We recommend utilising these modifiable biopsychosocial factors in future research and clinical practice to triage and match patients into targeted SM programmes.

## Supplementary Information

**Additional file 1:** **Table 1.** Demographic characteristics of the participants at baseline and comparison between completers and non-completers of the follow-up survey. **Table 2.** Characteristics of the participants at baseline. **Table 3.** Descriptive statistics for self-management constructs at baseline. **Table 4.** Spearman (rho) correlation for the self-management constructs at baseline. **Table 5.** Descriptive statistics after loss to follow up data imputation. **Figure 4.** Predictors of change in self-management constructs at follow up after mean substitution of the lost to follow up cases. **Figure 5.** Predictors of change in self-management constructs at follow up after last observation carried forward substitution of the lost to follow up cases.

## Data Availability

The datasets used and/or analysed during the current study are available from the corresponding author on reasonable request.

## Abbreviations

95% CI: 95% Confidence Interval
BCa: Bias Corrected and Accelerated
BOS: Bristol Online Survey
CLBP: Chronic low back pain
GLM: Generalised Linear Model
HDA: Health Directed Activity
heiQ: Health Education Impact Questionnaire
HSN: Health Service Navigation
IBM: International Business Machines Corporation
ICC: Intraclass Correlation Coefficient
IPAQ-SF: International Physical Activity Questionnaire- Short Form
NHS: National Health Service
NPS: Numeric Pain Scale
PCS: Pain Catastrophising Scale
PHQ-9: Patient Health Questionnaire-9
RMDQ: Roland Morris Disability Questionnaire
SD: Standard Deviation
SIS: Social Integration and Support
SM: Self-management
SMI: Self-Monitoring and Insight
YLDs: Years Lived with Disability
