# Supplementary material for: Predictors of self-management in patients with chronic low back pain: a longitudinal study
Source: BMC Musculoskelet Disord. 2022 Dec 7;23:1071. doi: 10.1186/s12891-022-05933-2 (PMC9727914; doi:10.1186/s12891-022-05933-2)
Supplement: Supplementary file 1 — Additional file 1: Table 1. Demographic characteristics of the participants at baseline and comparison between completers and non-completers of the follow-up survey. Table 2. Characteristics of the participants at baseline. Table 3. Descriptive statistics for self-management constructs at baseline. Table 4. Spearman (rho) correlation for the self-management constructs at baseline. Table 5. Descriptive statistics after loss to follow up data imputation. Figure 4. Predictors of change in self-management constructs at follow up after mean substitution of the lost to follow up cases. Figure 5. Predictors of change in self-management constructs at follow up after last observation carried forward substitution of the lost to follow up cases. [file 12891_2022_5933_MOESM1_ESM.docx]

**Supplementary Material (Additional Tables, Sensitivity analyses and STROBE Checklist)**

**Journal: BMC Musculoskeletal Disorders**

**Article title: Predictors of self-management in patients with chronic low back pain: a longitudinal study**

Table 1: Demographic characteristics of the participants at baseline and comparison between completers and non-completers of the follow-up survey

| Demographic characteristics | | All participants | | Non-completers | | Completers | |
| --- | --- | --- | --- | --- | --- | --- | --- |
|  | | number | percentage | number | percentage | number | percentage |
| Gender (n=269) | | | | | | | |
|  | Female | 165 | 61.1 | 70 | 26.0 | 95 | 35.3 |
|  | Male | 104 | 38.5 | 46 | 17.1 | 58 | 21.6 |
| Ethnicity (n=269) | | | | | | | |
|  | White | 226 | 84.1 | 95 | 35.3 | 131 | 48.7 |
|  | Black or Black British | 13 | 4.8 | 10 | 3.7 | 3 | 1.1 |
|  | Asian or Asian British | 16 | 5.9 | 5 | 1.9 | 11 | 4.1 |
|  | Mixed | 11 | 4.1 | 6 | 2.2 | 5 | 1.9 |
|  | Chinese | 1 | 0.4 | 0 | 0.0 | 1 | 0.4 |
|  | Other | 2 | 0.7 | 0 | 0.0 | 2 | 0.7 |
| The highest level of education (n=267)* | | | | | | | |
|  | No formal education | 1 | 0.4 | 1 | 0.4 | 0 | 0.0 |
|  | Primary school | 1 | 0.4 | 1 | 0.4 | 0 | 0.0 |
|  | Secondary school | 72 | 27.0 | 36 | 13.5 | 36 | 13.5 |
|  | High school | 18 | 6.7 | 10 | 3.7 | 8 | 3.0 |
|  | College/ professional | 77 | 28.8 | 38 | 14.2 | 39 | 14.6 |
|  | University | 98 | 36.7 | 30 | 11.2 | 68 | 25.5 |
| Marital status (n=268) | | | | | | | |
|  | Single | 81 | 30.3 | 42 | 15.7 | 39 | 14.6 |
|  | Married | 118 | 44.0 | 44 | 16.4 | 74 | 27.7 |
|  | Living as married | 34 | 12.7 | 15 | 5.6 | 19 | 7.1 |
|  | Widowed | 2 | 0.7 | 2 | 0.7 | 0 | 0.0 |
|  | Divorced/ separated | 28 | 10.4 | 11 | 4.1 | 17 | 6.3 |
|  | Other | 5 | 1.9 | 3 | 1.1 | 2 | 0.7 |
| Living arrangements (n=267) | | | | | | | |
|  | Living alone | 49 | 18.4 | 25 | 9.4 | 24 | 9.0 |
|  | Living with spouse or partner | 164 | 61.4 | 70 | 26.2 | 94 | 35.3 |
|  | Living with relative or friend | 38 | 14.2 | 15 | 5.6 | 23 | 8.6 |
|  | Living in shared accommodation | 9 | 3.4 | 3 | 1.1 | 6 | 2.2 |
|  | Others | 7 | 2.6 | 3 | 1.1 | 4 | 1.5 |
| Employment status (n=268) | | | | | | | |
|  | Retired | 14 | 5.2 | 3 | 1.1 | 11 | 4.1 |
|  | Student | 14 | 5.2 | 5 | 1.9 | 9 | 3.4 |
|  | Unemployed | 46 | 17.2 | 19 | 7.1 | 27 | 10.1 |
|  | Job searching | 4 | 1.5 | 2 | 0.7 | 2 | 0.7 |
|  | Working part-time | 53 | 19.8 | 22 | 8.2 | 31 | 11.6 |
|  | Working full-time | 137 | 51.1 | 65 | 24.3 | 72 | 26.9 |
| Annual household income (n=251) | | | | | | | |
|  | < £15,000 | 68 | 27.1 | 29 | 11.6 | 39 | 15.5 |
|  | £15,000-19,999 | 37 | 14.7 | 19 | 7.5 | 18 | 7.2 |
|  | £20,000-29,999 | 42 | 16.7 | 23 | 9.1 | 19 | 7.6 |
|  | £30,000-39,999 | 41 | 16.3 | 17 | 6.7 | 24 | 9.6 |
|  | £40,000-49,999 | 17 | 6.8 | 7 | 2.8 | 10 | 4.0 |
|  | £50,000-59,999 | 22 | 8.8 | 8 | 3.2 | 14 | 5.6 |
|  | £60,000-69,999 | 2 | 0.8 | 1 | 0.4 | 1 | 0.4 |
|  | £70,000-99,999 | 15 | 6.0 | 3 | 1.2 | 12 | 4.8 |
|  | £100,000-149,999 | 6 | 2.4 | 3 | 1.2 | 3 | 1.2 |
|  | £150,000+ | 1 | 0.4 | 0 | 0.0 | 1 | 0.4 |
| Living in 20% most deprived areas (n=269) | | | | | | | |
|  | Yes | 90 | 33.5 | 46 | 17.1 | 44 | 16.4 |
|  | No | 179 | 66.5 | 71 | 26.4 | 108 | 40.1 |

* Significant difference between the completers and non-completers, % calculated of the total sample

Table 2: Characteristics of the participants at baseline

| Variables | Number | Mean | SD | BCa 95% CI | |
| --- | --- | --- | --- | --- | --- |
|  |  |  |  | Lower | Upper |
| Age (year) | 270 | 43.74 | 11.89 | 42.30 | 45.19 |
| Pain duration (year) | 260 | 6.43 | 7.82 | 5.63 | 7.23 |
| NPS | 262 | 5.80 | 2.44 | 5.53 | 6.10 |
| RMDQ | 270 | 11.63 | 5.86 | 10.96 | 12.28 |
| PHQ | 268 | 8.68 | 6.41 | 7.86 | 9.51 |
| TSK | 269 | 38.73 | 7.43 | 37.91 | 39.65 |
| PCS | 269 | 18.10 | 13.00 | 16.57 | 19.58 |
| Pr. analgesic | 250 | 1.22 | 1.27 | 1.07 | 1.39 |
| OTC analgesic | 250 | 0.31 | 0.63 | 0.23 | 0.38 |
| Healthcare use | 258 | 5.34 | 5.52 | 4.68 | 6.14 |
| IMD | 269 | 4.55 | 2.95 | 4.22 | 4.91 |
| IPAQ | 268 | 2.92 | 3.68 | 2.48 | 3.40 |

N: sample size, SD: standard deviation, BCa 95%CI: bias corrected and accelerated 95% confidence interval, NPS: Numeric Pain Scale, RMDQ: Roland Morris Disability Questionnaire, PHQ: Patient Health Questionnaire, TSK: Tampa Scale of Kinesiophobia, PCS: Pain Catastrophising Scale, Pr. Analgesic: Number of prescribed analgesics, OTC analgesic: Number of Over the counter analgesic, IMD: Index for Multiple Deprivation, IPAQ: International Physical Activity Questionnaire (in Kilo Metabolic Equivalent)

Table 3: Descriptive statistics for self-management constructs at baseline

| Constructs (N=270) | Mean | SD | SEM | BCa 95% CI | |
| --- | --- | --- | --- | --- | --- |
|  |  |  |  | Lower | Upper |
| Health Directed Activity† | 2.87 | 0.66 | 0.04 | 2.78 | 2.95 |
| Positive and Active Engagement in Life† | 2.78 | 0.61 | 0.04 | 2.70 | 2.85 |
| Emotional Distress | 2.41 | 0.70 | 0.04 | 2.32 | 2.50 |
| Self-Monitoring and Insight | 2.98 | 0.45 | 0.03 | 2.92 | 3.03 |
| Constructive Attitudes and Approaches | 2.83 | 0.59 | 0.04 | 2.76 | 2.90 |
| Skill and Technique Acquisition | 2.64 | 0.54 | 0.03 | 2.57 | 2.70 |
| Social Integration and Support | 2.76 | 0.60 | 0.04 | 2.68 | 2.83 |
| Health Service Navigation | 2.86 | 0.50 | 0.03 | 2.80 | 2.92 |

N: sample size, SD: Standard Deviation, SEM: Standard Error of Mean, BCa: Bias Corrected and accelerated (for 1000 samples), 95%CI: 95% confidence interval, †N=269

Table 4: Spearman (rho) correlation for the self-management constructs at baseline

| Constructs | HDA | PAEL | ED | SMI | CAA | STA | SIS |
| --- | --- | --- | --- | --- | --- | --- | --- |
| Health Directed Activity (HDA) |  |  |  |  |  |  |  |
| Positive and Active Engagement in Life (PAEL) | 0.50** |  |  |  |  |  |  |
| Emotional Distress (ED) | 0.31** | 0.57** |  |  |  |  |  |
| Self-Monitoring and Insight (SMI) | 0.39** | 0.34** | 0.17** |  |  |  |  |
| Constructive Attitudes and Approaches (CAA) | 0.41** | 0.66** | 0.59** | 0.31** |  |  |  |
| Skill and Technique Acquisition (STA) | 0.35** | 0.47** | 0.34** | 0.54** | 0.48** |  |  |
| Social Integration and Support (SIS) | 0.28** | 0.44** | 0.26** | 0.32** | 0.50** | 0.42** |  |
| Health Service Navigation (HSN) | 0.41** | 0.36** | 0.15* | 0.50** | 0.37** | 0.53** | 0.46** |

** Correlation is significant at 0.01 level (2-tailed)

## **Sensitivity analyses using mean substitution and baseline observed carried forward data**

## **imputations for lost to follow up cases**

A total of 153 participants (56.7% of the recruited) completed the follow-up survey. There were no significant differences between the completers and non-completers of the follow-up survey except for the level of education. To examine the robustness of the main results, two sensitivity analyses were conducted using two data imputation algorithms for lost to follow up cases- follow up mean (mean substitution) and baseline carried forward (baseline observation carried forward) for each model variable. Table 6 summarises the mean and standard deviation of the SM constructs and biopsychological factors at follow up after the data imputations.

Multivariate regression was calculated using GLM to predict each of the SM constructs at follow up adjusted for their baseline values and based on their significant (*p*<.05) univariate predictor variables and categorical variables with significant differences (*p*<.05) for both the imputed datasets.

Figures 4 and 5 summarise the predictors of change in SM constructs based on the heiQ scores and the biopsychosocial factors after the mean substitution and baseline observation carried forward, respectively.

Results of these sensitivity analyses showed a difference in the variance (adjusted R^2^) of the predictive association between the SM constructs (or their changes) and the biopsychosocial factors, although the overall direction of the results supported the main results that the physical disability, depression, catastrophising and kinesiophobia predicted SM constructs and their change over time.

Table 5: Descriptive statistics after loss to follow up data imputation

| Mean substitution | N | Min | Max | Mean | SD | Last observed observation substitution | N | Min | Max | Mean | SD |
| --- | --- | --- | --- | --- | --- | --- | --- | --- | --- | --- | --- |
| NPS | 270 | 0.00 | 10.00 | 4.32 | 2.05 | NPS | 264 | 0.00 | 10.00 | 5.17 | 2.71 |
| RMDQ | 270 | 0.00 | 24.00 | 9.14 | 5.08 | RMDQ | 270 | 0.00 | 24.00 | 10.57 | 6.55 |
| HDA | 270 | 1.00 | 4.00 | 2.97 | 0.50 | HDA | 270 | 1.00 | 4.00 | 2.93 | 0.64 |
| PAEL | 270 | 1.20 | 4.00 | 2.88 | 0.47 | PAEL | 270 | 1.00 | 4.00 | 2.83 | 0.60 |
| ED | 270 | 1.00 | 4.00 | 2.38 | 0.55 | ED | 270 | 1.00 | 4.00 | 2.35 | 0.70 |
| CAA | 270 | 1.20 | 4.00 | 2.94 | 0.43 | CAA | 270 | 1.00 | 4.00 | 2.88 | 0.55 |
| SMI | 270 | 1.00 | 4.00 | 3.09 | 0.32 | SMI | 270 | 1.00 | 4.00 | 3.03 | 0.45 |
| SIS | 270 | 1.20 | 4.00 | 2.76 | 0.45 | SIS | 270 | 1.00 | 4.00 | 2.79 | 0.57 |
| STA | 270 | 1.50 | 4.00 | 2.79 | 0.39 | STA | 270 | 1.00 | 4.00 | 2.74 | 0.51 |
| HSN | 270 | 1.00 | 4.00 | 2.81 | 0.44 | HSN | 270 | 1.00 | 4.00 | 2.84 | 0.55 |
| PHQ | 270 | 0.00 | 26.00 | 7.86 | 5.32 | PHQ | 268 | 0.00 | 27.00 | 8.21 | 6.73 |
| TSK | 270 | 7.00 | 59.00 | 36.84 | 6.09 | TSK | 269 | 7.00 | 59.00 | 38.04 | 7.46 |
| PCS | 270 | 0.00 | 49.00 | 13.35 | 9.89 | PCS | 270 | 0.00 | 52.00 | 16.15 | 13.58 |
| IPAQ | 270 | 0.00 | 21.71 | 3.64 | 3.37 | IPAQ | 267 | 0.00 | 21.71 | 3.29 | 4.10 |
| Pr. Analgesic | 270 | 0.00 | 4.00 | 0.77 | 0.84 | Pr. Analgesic | 260 | 0.00 | 4.00 | 0.95 | 1.21 |
| OTC Analgesic | 270 | 0.00 | 3.00 | 0.33 | 0.44 | OTC Analgesic | 260 | 0.00 | 3.00 | 0.31 | 0.61 |
| Healthcare use | 270 | 0.00 | 32.00 | 1.89 | 2.64 | Healthcare use | 229 | 0.00 | 32.00 | 3.54 | 4.38 |

N: sample size, SD: standard deviation, Min: Minimum, Max: Maximum, NPS: Numeric Pain Scale, RMDQ: Roland Morris Disability Questionnaire, HDA: Health Directed Activity; PAEL: Positive and Active Engagement in Life; ED: Emotional Distress; SMI: Self-Monitoring and Insight; CAA: Constructive Attitudes and Approaches; STA: Skill and Technique Acquisition; SIS: Social Integration and Support; HSN: Health Service Navigation, PHQ: Patient Health Questionnaire, TSK: Tampa Scale of Kinesiophobia, PCS: Pain Catastrophising scale, Pr. Analgesic: Number of prescribed analgesics, OTC analgesic: Number of Over the counter analgesic, IMD: Index for Multiple Deprivation, IPAQ: International Physical Activity Questionnaire (in Kilo Metabolic Equivalent

B: unstandardised coefficient, *p*: *p*-value, Adj. R^2^: Adjusted R^2^, RMDQ: Roland Morris Disability Questionnaire; NPS: Numeric Pain Scale; PHQ: Patient Health Questionnaire-9; TSK: Tampa Scale of Kinesiophobia; PCS: Pain; Catastrophising scale; Pr. Analgesic: No of prescribed analgesics; OTC Analgesics: No of Over the Counter analgesics; IMD: Index for Multiple Deprivation; IPAQ: International Physical Activity Questionnaire (in Kilo Metabolic Equivalent; Healthcare use: No. of visits to healthcare providers in last 3 months; HDA: Health Directed Activity; PAEL: Positive and Active Engagement in Life; ED: Emotional Distress; SMI: Self-Monitoring and Insight; CAA: Constructive Attitudes and Approaches; STA: Skill and Technique Acquisition; SIS: Social Integration and Support; HSN: Health Service Navigation, Married: Married and living with partner,

Living as married: Living with spouse or partner, Employed: Full- or part-time employed, High income: Household income >£30000 in a year, College: Highest level of education at college or university, treatment type; Physiotherapy only or pain management only, White: White ethnic background, Gender: Female vs. male

Figure 4: Predictors of change in self-management constructs at follow up after mean substitution of the lost to follow up cases

B: unstandardised coefficient, *p*: *p*-value, Adj. R^2^: Adjusted R^2^, RMDQ: Roland Morris Disability Questionnaire; NPS: Numeric Pain Scale; PHQ: Patient Health Questionnaire-9; TSK: Tampa Scale of Kinesiophobia; PCS: Pain; Catastrophising scale; Pr. Analgesic: No of prescribed analgesics; OTC Analgesics: No of Over the Counter analgesics; IMD: Index for Multiple Deprivation; IPAQ: International Physical Activity Questionnaire (in Kilo Metabolic Equivalent; Healthcare use: No. of visits to healthcare providers in last 3 months; HDA: Health Directed Activity; PAEL: Positive and Active Engagement in Life; ED: Emotional Distress; SMI: Self-Monitoring and Insight; CAA: Constructive Attitudes and Approaches; STA: Skill and Technique Acquisition; SIS: Social Integration and Support; HSN: Health Service Navigation, Married: Married and living with partner,

Living as married: Living with spouse or partner, Employed: Full- or part-time employed, High income: Household income >£30000 in a year, College: Highest level of education at college or university, treatment type; Physiotherapy only or pain management only, White: White ethnic background, Gender: Female vs. male

Figure 5: Predictors of change in self-management constructs at follow up after last observation carried forward substitution of the lost to follow up cases

**STROBE Statement—Checklist of items that should be included in reports of *cohort studies***

[Available from <https://www.equator-network.org/reporting-guidelines/strobe/>, last accessed 29 May 2022)]

|  | Item No | Recommendation | Page No |
| --- | --- | --- | --- |
| **Title and abstract** | 1 | (*a*) Indicate the study’s design with a commonly used term in the title or the abstract | Yes, 1 |
|  |  | (*b*) Provide in the abstract an informative and balanced summary of what was done and what was found | Yes, 1 |
| Introduction | | | |
| Background/rationale | 2 | Explain the scientific background and rationale for the investigation being reported | Yes, 1-2 |
| Objectives | 3 | State specific objectives, including any prespecified hypotheses | Yes, 2 |
| Methods | | | |
| Study design | 4 | Present key elements of study design early in the paper | Yes, 2-3 |
| Setting | 5 | Describe the setting, locations, and relevant dates, including periods of recruitment, exposure, follow-up, and data collection | Yes, 2-3 |
| Participants | 6 | (*a*) Give the eligibility criteria, and the sources and methods of selection of participants. Describe methods of follow-up | Yes, 2-3 |
|  |  | (*b*) For matched studies, give matching criteria and number of exposed and unexposed | Not applicable |
| Variables | 7 | Clearly define all outcomes, exposures, predictors, potential confounders, and effect modifiers. Give diagnostic criteria, if applicable | Yes, 2-3 |
| Data sources/ measurement | 8* | For each variable of interest, give sources of data and details of methods of assessment (measurement). Describe comparability of assessment methods if there is more than one group | Yes, 2-3 |
| Bias | 9 | Describe any efforts to address potential sources of bias | Yes, 2-3 |
| Study size | 10 | Explain how the study size was arrived at | Yes, 2-3 |
| Quantitative variables | 11 | Explain how quantitative variables were handled in the analyses. If applicable, describe which groupings were chosen and why | Yes, 2-3 |
| Statistical methods | 12 | (*a*) Describe all statistical methods, including those used to control for confounding | Yes, 2-3 |
|  |  | (*b*) Describe any methods used to examine subgroups and interactions | Yes, Supplementary files |
|  |  | (*c*) Explain how missing data were addressed | Yes, Supplementary files |
|  |  | (*d*) If applicable, explain how loss to follow-up was addressed | Yes, Supplementary files |
|  |  | (*e*) Describe any sensitivity analyses | Yes, Supplementary files |
| Results | | |  |
| Participants | 13* | (a) Report numbers of individuals at each stage of study—eg numbers potentially eligible, examined for eligibility, confirmed eligible, included in the study, completing follow-up, and analysed | Yes, 4 |
|  |  | (b) Give reasons for non-participation at each stage | Yes, Figure 1 |
|  |  | (c) Consider use of a flow diagram | Yes, Figure 1 |
| Descriptive data | 14* | (a) Give characteristics of study participants (eg demographic, clinical, social) and information on exposures and potential confounders | Yes, Supplementary file and page 4 |
|  |  | (b) Indicate number of participants with missing data for each variable of interest | Yes, Supplementary file |
|  |  | (c) Summarise follow-up time (eg, average and total amount) | Yes, Supplementary file and page 4 |
| Outcome data | 15* | Report numbers of outcome events or summary measures over time | Yes, Page 4 |

| Main results | 16 | (*a*) Give unadjusted estimates and, if applicable, confounder-adjusted estimates and their precision (eg, 95% confidence interval). Make clear which confounders were adjusted for and why they were included | Yes, Page 4 |
| --- | --- | --- | --- |
|  |  | (*b*) Report category boundaries when continuous variables were categorized | Yes, Page 4 |
|  |  | (*c*) If relevant, consider translating estimates of relative risk into absolute risk for a meaningful time period | Not applicable |
| Other analyses | 17 | Report other analyses done—eg analyses of subgroups and interactions, and sensitivity analyses | Yes, Supplementary file |
| Discussion | | | |
| Key results | 18 | Summarise key results with reference to study objectives | Yes, Page 5 |
| Limitations | 19 | Discuss limitations of the study, taking into account sources of potential bias or imprecision. Discuss both direction and magnitude of any potential bias | Yes, Page 6 |
| Interpretation | 20 | Give a cautious overall interpretation of results considering objectives, limitations, multiplicity of analyses, results from similar studies, and other relevant evidence | Yes, Page 6 |
| Generalisability | 21 | Discuss the generalisability (external validity) of the study results | Yes, Page 6 |
| Other information | | | |
| Funding | 22 | Give the source of funding and the role of the funders for the present study and, if applicable, for the original study on which the present article is based | Yes, Page 6 |

*Give information separately for exposed and unexposed groups.

**Note:** An Explanation and Elaboration article discusses each checklist item and gives methodological background and published examples of transparent reporting. The STROBE checklist is best used in conjunction with this article (freely available on the Web sites of PLoS Medicine at http://www.plosmedicine.org/, Annals of Internal Medicine at http://www.annals.org/, and Epidemiology at http://www.epidem.com/). Information on the STROBE Initiative is available at http://www.strobe-statement.org.
